# Wrapping pancreaticojejunostomy using the ligamentum teres hepatis during laparoscopic pancreaticoduodenectomy: a propensity score matching analysis

**DOI:** 10.1186/s12957-023-03255-8

**Published:** 2023-11-18

**Authors:** Jia-Guo Wang, Kai Lei, Ke You, Jie Xu, Zuo-Jin Liu

**Affiliations:** Department of Hepatobiliary Surgery, The Second Affiliated Hospital of Chongqing Medical University, Yuzhong District, Chongqing, 400010 China

**Keywords:** Postoperative pancreatic fistula, Laparoscopic pancreaticoduodenectomy, Ligamentum teres hepatis, Pancreaticojejunostomy, Propensity score matching analysis

## Abstract

**Background and Objective:**

It is controversial whether wrapping around the pancreaticojejunostomy (PJ) could reduce the rate of postoperative pancreatic fistula (POPF), especially in laparoscopic pancreaticoduodenectomy (LPD). This study aims to summarize our single-center initial experience in wrapping around PJ using the ligamentum teres hepatis (LTH) and demonstrate the feasibility and safety of this method.

**Methods:**

Patients who underwent LPD applying the procedure of wrapping around the PJ were identified. The cohort was compared to the cohort with standard non-wrapping PJ. A 1:1 propensity score matching (PSM) was performed to compare the early postoperative outcomes of the two cohorts. Risk factors for POPF were determined by using univariate and multivariate logistic regression analysis.

**Results:**

Overall, 143 patients were analyzed (LPD without wrapping (*n* = 91) and LPD with wrapping (*n* = 52)). After 1:1 PSM, 48 patients in each cohort were selected for further analysis. Bile leakage, DGE, intra-abdominal infection, postoperative hospital stays, harvested lymph nodes, and R0 resection were comparable between the two cohorts. However, the wrapping cohort was associated with significantly less POPF B (1 vs 18, *P* = 0.003), POPF C (0 vs 8, *P* = 0.043), and Clavien–Dindo classification level III–V (5 vs 26, *P* = 0.010). No patients died due to the clinically relevant POPF in the two cohorts. No patients who underwent the LTH wrapping procedure developed complications directly related to the wrapping procedure. After PSM, whether wrapping was an independent risk factor for POPF (OR = 0.202; 95%CI:0.080–0.513; *P* = 0.001).

**Conclusions:**

Wrapping the LTH around the PJ technique for LPD was safe, efficient, and reproducible with favorable perioperative outcomes in selected patients. However, further validations using high-quality RCTs are still required to confirm the findings of this study.

**Supplementary Information:**

The online version contains supplementary material available at 10.1186/s12957-023-03255-8.

## Introduction

With advances in surgical techniques and perioperative management, surgical-relevant mortality of LPD has decreased to below 4% in a high-volume center, but the incidence of POPF is between 14 and 26% [1–4]. POPF always acts as the major determinant of morbidity and mortality after LPD, developing serious postoperative complications [5]. Various methods, including various surgical techniques and medical treatments (e.g., somatostatin analogues), have been tried to reduce the risk of POPF and its sequelae [6], but none of the above methods was shown to be fully effective.

As early as 1994, the use of omentum or falciform ligament for local retroperitoneal vessels and/or PJ was first reported [7]. In recent years, with the popularization of this technology, a growing number of studies suggested that it is controversial whether wrapping around the PJ could significantly reduce the rate of POPF in open pancreaticoduodenectomy (OPD) [8, 9]. Iannitti, D.A., et al., first described that using round ligaments as vascular pedicles to strengthen pancreatic anastomosis decreases the rate of POPF [10]. However, to the best of our knowledge, the methods described above are only used for open surgery.

With the aim of reducing the rate of POPF, and considering the simplicity and reproducible of the wrapping technology, we have adopted the wrapping technology to our surgical procedure, The aim of this study was to provide our initial experience using the LTH wrapping of pancreatic anastomosis and assess whether or not the use of this method could reduce the rate of POPF and PPH in patients who undergo LPD.

## Methods

### Patients

The patients who underwent LPD applying the procedure of wrapping around the PJ in the Second Affiliated Hospital of Chongqing Medical University between January 2018 and December 2022 were retrospectively analyzed. Of these, we have started to routinely use the LTH to wrap around the PJ after November 2021, while this method has not been done before. Preoperative biochemical and imaging examinations (CT/MRI) were routinely performed in all patients, and all clinical data were collected retrospectively. Prophylactic antibiotic therapy was intravenously administered 30 min before surgery and maintained until the seventh postoperative day for regular patient, the type, dose and course of antibiotic therapy will be adjusted according to the real-time changes in patients’ condition. Warm glucose saline was slowly injected through the gastric tube on the first day, and “nourishing enteral nutrition” was started on the third day under the guidance of a clinical dietitian. Post-operative management included hematischesis, inhibition of pancreatic enzymes, rehydration, acid suppression and stomach protection, analgesia and other symptomatic and supportive care.

All individual participant included in this study had signed informed consent for reviewing and researching their anonymized clinical data. This study has been approved by the Ethics Committee of the Second Affiliated Hospital of Chongqing Medical University.

### Perioperative data collection and Definitions

POPF [5], delayed gastric emptying (DGE) [11], and PPH [12] were defined according to the International Standard of Research Group of Pancreatic Fistula (ISGPF).

The following variables were retrospectively reviewed and analyzed: 1) The preoperative data included age, gender, body mass index (BMI), comorbidity, routine preoperative laboratory examination, Pancreatic CT value, pancreatic tube diameter and American Society of Anesthesiologists (ASA) score. The intraoperative data comprised information on the length of operative time, blood loss. The postoperative data mainly included postoperative complications PPH, POPF, biliary leakage, diarrhoea and DGE, the Clavien–Dindo classification, intra-abdominal infection, bowel obstruction, 30-day mortality, R0 resection, harvested lymph nodes and positive lymph nodes.

### Surgical techniques for wrapping of the PJ

**Fig. 1:**
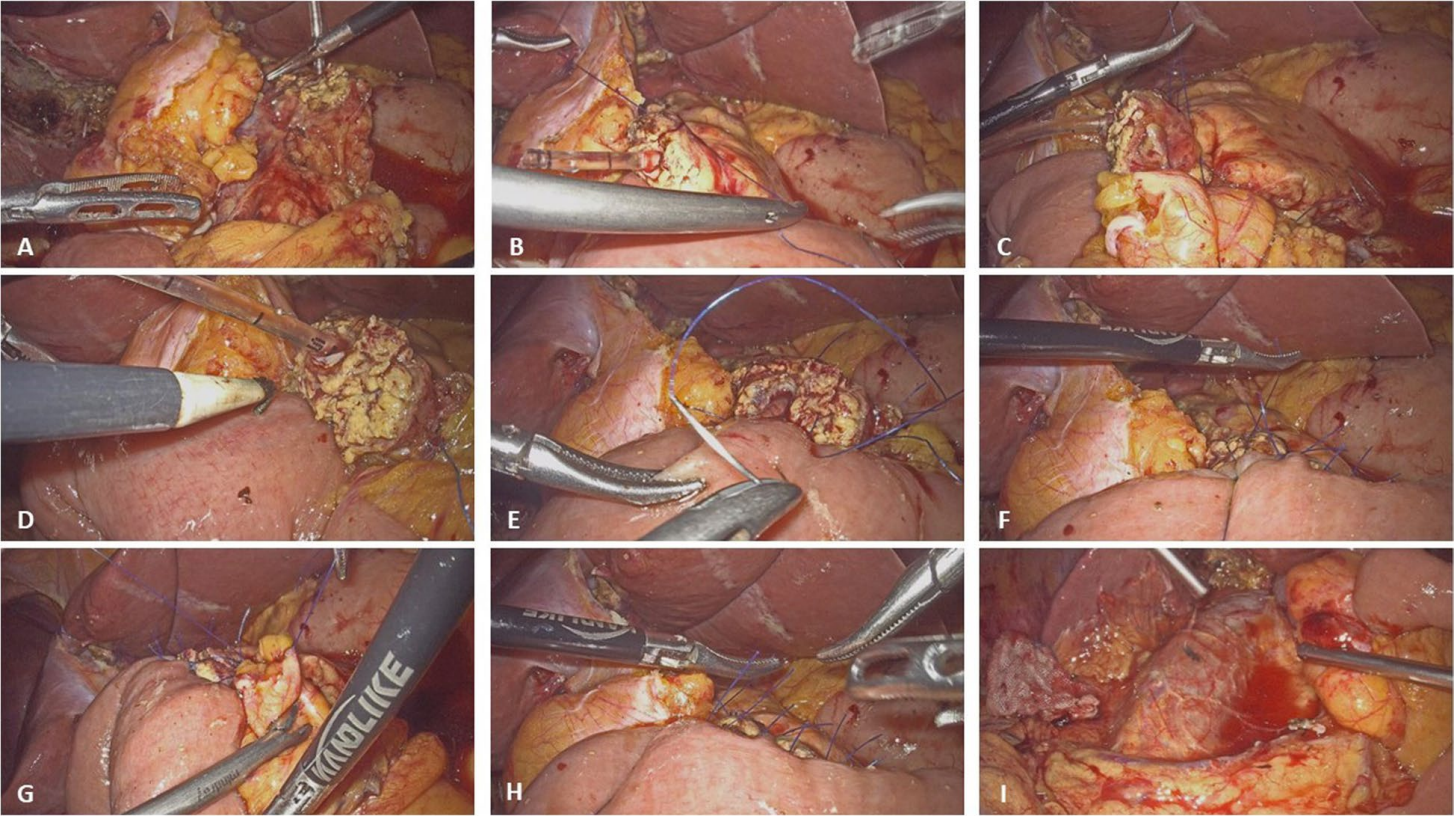
Wrapping technique of the PJ. **A** The pancreatic stump and the LTH stump were exposed in the visual field. **B, C** The modified Blumgart’s method used two transpancreatic-LTH- jejunal seromuscular U-shaped sutures to approximate the pancreas, LTH and the jejunum. The LTH was fixed behind the pancreatoenteric anastomosis. **D** The location of the pancreatoenteric anastomosis was marked on the jejunum. **E** The duct-to-mucosa PJ technique was used to draining pancreatic juice into the intestinal lumen. **F, G** The LTH was used to cover the upper and inferior margin of pancreatoenteric anastomosis. **H, I** The ventral and dorsal view of the wrapped pancreatoenteric anastomosis

**Fig. 2:**
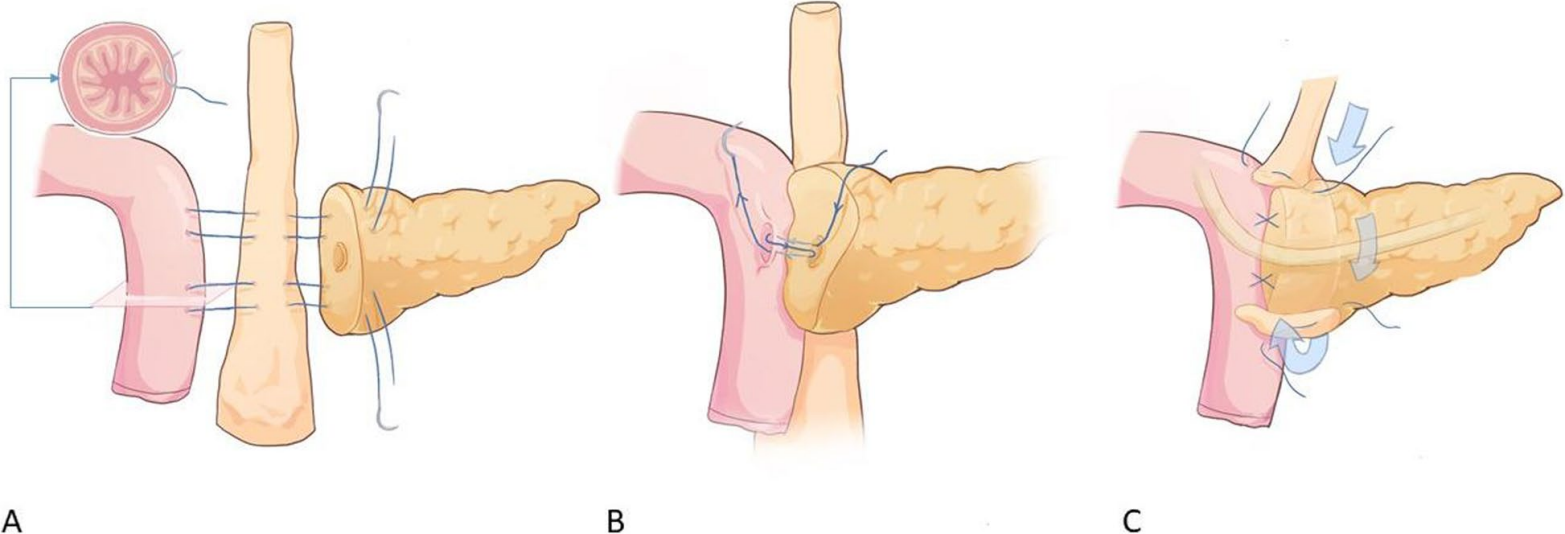
Diagram of wrapping PJ technique. **A** Two transpancreatic-LTH- jejunaluscular U-shaped sutures were employed to approximate the pancreas and jejunum, with the LTH serving as a pad for wrapping the posterior wall of the PJ. **B** Three to five transpancreatic—duct—jejunum full-thickness figure-eight sutures was used to complete the duct-to-mucosa PJ. **C** One transpancreatic-LTH- jejunaluscular interrupted suture was used to cover the superior and inferior margin of PJ separately

The pancreatic stump was exposed in the visual field, and the LTH was mobilized around the pancreatic stump (Fig. 1A). A silicone catheter was inserted into the main pancreatic duct as an internal stent. The modified Blumgart’s method [13] used two transpancreatic-LTH-jejunal seromuscular U-shaped sutures to approximate the pancreas, LTH and the jejunum. The LTH was fixed behind the pancreatoenteric anastomosis (Fig. 1B, C). a hole was created in the jejunum using the electronic coagulator (Fig. 1D), and the other end of the silicone tube was inserted into the lumen of the jejunal intestine. The figure-eight suture was carried out for the posterior wall of the anastomosis between the posterior wall of the main pancreatic duct and the full layer of the jejunu, and this layer used only two to four sutures. the anterior wall of anastomosis was completed between the anterior wall of the main pancreatic duct and the anterior wall of the jejunum, and this layer used three to five sutures using same suture manner (Fig. 1E). The LTH was used to cover the upper and inferior margin of pancreatoenteric anastomosis (Fig. 1F, G). The ventral and dorsal view of the wrapped pancreatoenteric anastomosis (Fig. 1H, I). Diagram of wrapping PJ technique is shown in Fig. 2.

### Propensity score matching analysis

Propensity score matching analysis was performed to eliminate confounding variables between the two cohorts. This analysis matched variables that were significantly different between the twocohorts and variables that might have an impact on the postoperative outcome, including CEA, pancreatic CT value, and pancreatic tube diameter. Numerous studies have been conducted on the CT value of the pancreas as an objective indicator of pancreatic texture (firm or soft), rather than relying on subjective evaluations, the CT value of the pancreas and the diameter of the pancreatic duct were closely related to the occurrence of POPF [14–16], so we performed PSM. CEA varied between the groups in this study, and to eliminate its interference with the study, we also performed PSM. Furthermore, it is noteworthy that all surgical procedures were conducted by the same surgeon, all patients had pancreatic duct stenting, uniform suture methods were employed throughout, and there was no significant statistical difference between SEX and BMI between the two cohorts. Consequently, these variables were excluded from PSM in our study. A matching caliper of 0.02 and 1:1 nearest neighbor matching was used in this matching analysis.

### Statistical analysis

This study used SPSS 26.0 software (IBM, Chicago, IL, USA). Baseline data, Intraoperative variables, and Postoperative variables between the two cohorts were performed by using descriptive statistics. Mean and standard deviation (SD) were used to describe the variables meeting the normal distribution. Variables that did not fit the normal distribution were described by using the median and interquartile range (IQR). Categorical variables were summarized by using counts and percentages. Before PSM, comparisons between the two cohorts were finished by using the independent samples t-test to compare parametric variables, using the Mann–Whitney U test to compare nonparametric variables, and using the Chi-square test to compare categorical variables. After PSM, comparisons between the two cohorts were finished by using the paired t-test to compare parametric variables, using the Wilcoxon rank-sum test to compare nonparametric variables, and using the MeNemar test to compare categorical variables. Univariate and multivariate logistic regression analyses were performed to identify the independent predictors of POPF. A *P* value less than 0.05 was defined as statistical significance.

## Results

### Baseline characteristics

Given that the safety and feasibility of this technique can be observed in the short term, a minimum of 30 days of follow-up provides enough time to make the surgical complications significant and reduce the loss to follow-up. We successfully wrapped the pancreatoenteric anastomosis in 51 patients (Wrapping cohort) who underwent LPD after November 2021, 92 patients (the non-wrapping cohort) did not wrap around pancreatoenteric anastomosis between January 2018 and November 2021. Baseline characteristics of all patients are summarized before PSM in Table 1. The two cohorts differed before PSM in terms of carcinoembryonic antigen (CEA) (*P* = 0.028) and pancreatic CT value (*P* = 0.026). Baseline characteristics of all patients are summarized after PSM in Table 2. 48 patients in each cohort were well-matched and the baseline demographics were comparable.

**Table 1:** Patient characteristics before propensity score matching

Variables		Non-Wrapping Cohort (*n* = 92)	Wrapping Cohort (*n* = 51)	*P* value
**Sex**	**Female**	33(35.9%)	21(41.2%)	0.531
	**Male**	59(64.1%)	30(58.8%)	
**Age (years)**		61(13)	65(10)	0.053
**BMI (kg/m**^**2**^**)**		22.0(20.3–24.4)	21.3(19.6–22.9)	0.150
**Hypertension**		26(28.3%)	12(23.5%)	0.540
**Diabetes**		15(16.3%)	4(7.8%)	0.153
**Primary disease**				
**Pancreatic ductal adenocarcinoma**		41(44.6%)	27(52.9%)	
**Ampullary carcinoma**		14(15.2%)	9(17.6%)	
**Adenocarcinoma of the duodenum**		22(23.9%)	8(15.7%)	0.737
**Cholangiocarcinoma**		12(13.0%)	5(9.8%)	
**Chronic pancreatitis**		3(3.3%)	2(3.9%)	
**Liver function**				
**ALT (U/L)**		83(32–187)	117(28–235)	0.307
**AST (U/L)**		54(27–122)	68(23–179)	0.479
**TBIL (umol/L)**		58.9(10.1–161.9)	69.0(13.6–174.2)	0.574
**ALB (g/L)**		37.9(35.1–40.7)	38.6(34.2–42.9)	0.465
**Tumor markers**				
**CA-199(U/mL)**		54.59(14.79–318.0)	108.90(14.71–438.0)	0.387
**CA-125(U/mL)**		17.35(10.98–28.68)	21.70(9.38–34.40)	0.606
**CEA (ng/mL)**		1.95(0.97–2.96)	2.51(1.47–4.21)	**0.028**
**Pancreatic CT value (Hu)**		37(31–42)	34(29–39)	**0.026**
**Main pancreatic diameter(mm)**		3.51(2.53–4.78)	3.91(2.50–5.30)	0.572
**ASA classification**	**I**	1(1.1%)	0	0.447
	**II**	45(48.9%)	20(39.2%)	
	**III**	45(48.9%)	31(60.8%)	
	**IV**	1(1.1%)	0	

*ALB *Albumin, *CA *Carbohydrate antigen, *CEA *Carcinoembryonic antigen, *CT *Computed tomography, *ASA *American society of anesthesiologists

**Table 2:** Patient characteristics after propensity score matching

Variables		Non-Wrapping Group (*n* = 48)	Wrapping Group (*n* = 48)	*P* value
**Sex**	**Female**	16(33.3%)	20(41.7%)	0.399
	**Male**	32(66.7%)	28(58.3%)	
**Age (years)**		62(13)	65(10)	0.156
**BMI (kg/m**^**2**^**)**		21.27(19.00–23.81)	21.20(19.55–22.67)	0.750
**Hypertension**		13(27.1%)	10(20.8%)	0.473
**Diabetes**		8(16.7%)	3(6.3%)	0.109
**Primary disease**				
**Pancreatic ductal adenocarcinoma**		22(45.8%)	26(54.2%)	
**Ampullary carcinoma**		7(14.6%)	8(16.7%)	
**Adenocarcinoma of the duodenum**		15(31.3%)	8(16.7%)	0.557
**Cholangiocarcinoma**		3(6.3%)	4(8.3%)	
**Chronic pancreatitis**		1(2.1%)	2(4.2%)	
**Liver function**				
**ALT (U/L)**		88(38–197)	125(28–250)	0.468
**AST (U/L)**		69(30–130)	72(25–181)	0.679
**TBIL (umol/L)**		75.2(10.2–165.3)	72.0(13.7–178.3)	0.817
**ALB (g/L)**		36.4(33.8–39.3)	38.7(34.2–42.8)	0.057
**Tumor markers**				
**CA-199(U/mL)**		61.45(19.49–442.85)	102.53(14.74–411.2)	0.918
**CA-125(U/mL)**		23.40(11.40–34.85)	23.40(9.42–34.48)	0.866
**CEA (ng/mL)**		2.24(1.58–3.26)	2.57(1.50–4.30)	0.364
**Pancreatic CT value (Hu)**		35(30–40)	34(31–40)	0.912
**Main pancreatic diameter(mm)**		4.08(3.05–5.47)	3.95(2.63–5.35)	0.644
**ASA classification**	**I**	1(2.1%)	0	0.534
	**II**	20(41.7%)	19(39.6%)	
	**III**	26(54.2%)	29(60.4%)	
	**IV**	1(2.1%)	0	

*ALB *Albumin, *CA *Carbohydrate antigen, *CEA *Carcinoembryonic antigen, *CT *Computed tomography, *ASA *American society of anesthesiologists

### Postoperative outcomes

The comparison of the intraoperative and postoperative short-term outcomes between the two cohorts is shown before and after PSM in Tables 3 and 4, respectively. After PSM, regarding the intraoperative outcomes, no significant differences were noted in operative time between the two cohorts. The non-wrapping cohort was associated with significantly more intraoperative blood compared to the wrapping cohort (400.0 vs 200.0 min, *P* < 0.001).

**Table 3:** Intraoperative data and postoperative outcomes before PSM

Variables		Non-Wrapping Group (*n* = 92)	Wrapping Group (*n* = 51)	*P* value
**Operation time (min)**		400(351–469)	395(345–445)	0.183
**Blood loss (ml)**		300(200–600)	200(100–300)	** < 0.001**
**PPH**		20(21.7%)	5(9.8%)	0.068
**PPH A**		17(18.5%)	5(9.8%)	0.168
**PPH B**		2(2.2%)	0	0.289
**PPH C**		1(1.1%)	0	0.455
**POPF**		42(45.7%)	7(13.7%)	** < 0.001**
	**POPF A**	17(18.5%)	6(11.8%)	0.295
	**POPF B**	18(19.6%)	1(2.0%)	**0.003**
	**POPF C**	7(7.6%)	0	**0.043**
**Bile leakage**		12(13.0%)	2(3.9%)	0.079
**DGE**		12(13.0%)	8(15.7%)	0.663
**Diarrhoea**		4(4.3%)	11(21.6%)	0.253
**Clavien–Dindo I-II**		20(21.7%)	12(23.5%)	0.806
**Clavien–Dindo III–V**		26(28.3%)	5(9.8%)	**0.010**
**Intra-abdominal infection**		42(45.7%)	16(31.4%)	0.096
**Bowel obstruction**		6(6.5%)	1(2.0%)	0.226
**Time to remove first drainage tube (days)**		7(6–9)	8(6–8)	0.258
**Time to remove all drainage tube (days)**		14(10–20)	12(10–16)	0.281
**PHS (days)**		16(12–21)	14(12–19)	0.199
**Tumor source**	**Pancreas**	41(44.6%)	27(52.9%)	0.737
	**Ampulla**	14(15.2%)	9(17.6%)	
	**Duodenum**	22(23.9%)	8(15.7%)	
	**Bile duct**	12(13.0%)	5(9.8%)	
	**Other**	3(3.3%)	2(3.9%)	
**R0 resection**		91(98.9%)	50(98.0%)	0.670
**Harvested lymph nodes**		14(11–18)	16(12–21)	**0.034**
**Lymphatic metastasis**		0.34(0.98)	0.47(1.19)	0.470

*POPF* Postoperative pancreatic fistula, *DGE *Delayed gastric emptying, *PPH *Postpancreatectomy hemorrhage, *PHS *Postoperative hospital stays

**Table 4:** Intraoperative data and postoperative outcomes after PSM

Variables		Non-Wrapping Group (*n* = 48)	Wrapping Group (*n* = 48)	*P* value
**Operation time (min)**		400(343–479)	393(339–444)	0.244
**Blood loss (ml)**		400(200–600)	200(125–306)	** < 0.001**
**PPH**		11(22.9%)	5(10.4%)	0.100
**PPH A**		9(18.8%)	5(10.4%)	0.247
**PPH B**		2(4.2%)	0	0.153
**PPH C**		0	0	1.000
**POPF**		18(37.5%)	7(14.6%)	**0.011**
	**POPF A**	7(14.6%)	6(12.5%)	0.765
	**POPF B**	5(10.4%)	1(2.1%)	**0.050**
	**POPF C**	5(10.4%)	0	**0.022**
**Bile leakage**		5(10.4%)	2(4.2%)	0.239
**DGE**		6(12.5%)	7(14.6%)	0.765
**Diarrhoea**		5(10.4%)	9(18.8%)	0.247
**Clavien–Dindo I-II**		10(20.8%)	11(22.9%)	0.805
**Clavien–Dindo III–V**		16(33.3%)	5(10.4%)	**0.007**
**Intra-abdominal infection**		19(39.6%)	16(33.3%0	0.525
**Bowel obstruction**		4(8.3%)	1(2.1%)	0.168
**Time to remove first drainage tube (days)**		7(6–9)	8(6–8)	0.306
**Time to remove all drainage tube (days)**		14(10–20)	12(10–17)	0.690
**PHS (days)**		15(12–23)	14(12–19)	0.394
**Tumor source**	**Pancreas**	22(45.8%)	26(54.2%)	0.557
	**Ampulla**	7(14.6%)	8(16.7%)	
	**Duodenum**	15(31.3%)	8(16.7%)	
	**Bile duct**	3(6.3%)	4(8.3%)	
	**Other**	1(2.1%)	2(4.2%)	
**R0 resection**		48(100%)	47(97.9%)	0.315
**Harvested lymph nodes**		15(12–18)	17(12–21)	0.085
**Lymphatic metastasis**		0.50(1.27)	0.50(1.22)	1.000

*POPF *Postoperative pancreatic fistula, *DGE *Delayed gastric emptying, *PPH *Postpancreatectomy hemorrhage, *PHS *Postoperative hospital stays

Regarding the postoperative outcomes, bile leakage, DGE, diarrhoea, intra-abdominal infection, harvested lymph nodes, tumor source, and R0 resection were comparable between the two cohorts. The Clavien–Dindo classification level I-II and POPF A showed no statistical significance. The wrapping cohort was associated with significantly less POPF B (1 vs 18, *P* = 0.003), POPF C (0 vs 8, *P* = 0.043) and Clavien–Dindo classification level III–V (5 vs 26, *P* = 0.010) than the non-wrapping cohort. Although no statistical significance, we found that POPF of grade B and C occurred more frequently in non-wrapping cohort, resulting in a longer time to remove all drainage tubes in non-wrapping cohort (14 vs 12, *P* = 0.690). The first drainage tube was usually located around the gastrointestinal anastomosis and therefore no differences were observed in time to remove first drainage tube (7 vs 8, *P* = 0.306). Although not statistically significant, the incidence of PPH was higher in non-wrapping cohort (11 vs 5, *P* = 0.100) may be related to higher POPF of grade B and C, while postoperative hospital stays were longer in non-wrapping cohort (15 vs 14, *P* = 0.394). No patients died due to the clinically relevant POPF in the two cohorts. No patients who underwent the LTH wrapping procedure developed complications directly related to the wrapping procedure.

### Univariable and multivariable logistic regression analyses of POPF after PSM

After PSM, univariate and multivariate logistic regression analyses were used to assess the effect of variables on the occurrence of POPF (Table 5). After PSM, whether wrapping was an independent risk factor for POPF (OR = 0.202; 95%CI:0.080–0.513; *P* = 0.001).

**Table 5:** Univariable and multivariable logistic regression analyses of POPF after PSM

Variables	Univariate analysis		Multivariate analysis	
	**OR (95%CI)**	**P value**	**OR (95%CI)**	**P value**
**Sex**	1.776(0.658,4.793)	0.257		
**Age (years)**	0.968(0.941,0.997)	**0.029**	0.990(0.959,1.023)	0.556
**BMI (kg/m**^**2**^**)**	1.118(1.007,1.241)	**0.036**	1.130(1.006,1.269)	**0.039**
**Hypertension (Yes)**	0.718(0.321,1.609)	0.421		
**Diabetes (Yes)**	0.869(0.309,2.449)	0.791		
**Liver function**				
**ALT (U/L)**	1.001(0.998,1.003)	0.466		
**AST (U/L)**	0.999(0.995,1.003)	0.781		
**TBIL (umol/L)**	1.001(0.998,1.005)	0.532		
**ALB (g/L)**	1.012(0.941,1.088)	0.750		
**Tumor markers**				
**CA-199(U/mL)**	1.000(0.999,1.001)	0.987		
**CA-125(U/mL)**	1.002(0.996,1.008)	0.435		
**CEA (ng/mL)**	1.016(0.925,1.115)	0.744		
**Pancreatic CT value (Hu)**	1.028(0.988,1.069)	0.175		
**Main pancreatic diameter (mm)**	0.855(0.704,1.038)	0.113		
**ASA classification**	0.430(0.218,0.847)	**0.015**	0.432(0.198,0.945)	**0.036**
**Wrapping (Yes)**	0.189(0.077,0.464)	** < 0.001**	0.202(0.080,0.513)	**0.001**

*POPF *Postoperative pancreatic fistula, *ALB *Albumin, *CA *Carbohydrate antigen, *CEA *Carcinoembryonic antigen, *CT *Computed tomography, *ASA *American Society of Anesthesiologists

## Discussion

The prevention of POPF is also a major concern for every pancreatic surgeon in LPD. Although there are many studies on how to reduce POPF [6], none of these studies has fully confirmed that it is effective for prevention of POPF, and the subjects of these studies are open pancreaticoduodenectomy. To the best of our knowledge, the use of the LTH to wrap anastomosis was the first described during LPD. Our study revealed that the LTH wrapping significantly reduced complications of Clavien–Dindo classification level III–V, especially POPF of grade B and C, while it did not increase the operation time and the difficulty of the operation. no patients who underwent the LTH wrapping procedure developed complications directly related to the wrapping procedure.

The falciform ligament and omental flaps have been used to wrap the pancreatoenteric anastomosis during open pancreaticoduodenectomy [8, 17–19]. The falciform ligament and omental are by the advantage of neovascularization, defense against infections, excellent blood supply and great capabilities for fluids absorption and adhesion formation [17, 19], which promote to heal the anastomosis through adhesion and granulation tissue formation. From our initial experience, our wrapping technique of anastomosis, which includes falciform ligament flap preparation, mobilization, and suturing, can be easily completed and standardized (Fig. 1, Additional file 1), and do not prolong excessive operation time. Secondly, compared with the interrupted suture of duct-to-mucosa anastomosis, The advantages of the "figure-eight suture" are as follows: 1) it can prevent inadequate tissue suture, which may lead to ineffective suture; 2) it is a simple and secure technique with fewer knotting times; 3) based on our preliminary clinical experience, the incidence of anastomotic fistula or stenosis in this way is not inferior to that of interrupted suture. Third, regarding the " Wrapping was performed centered on the posterior wall”. it is a matter of the length of the LTH, the main pancreatic duct is near the dorsal pancreas, and the posterior wall of the anastomosis is relatively weak. Therefore, we prefer to strengthen the posterior wall with LTH.

Several studies have reported that a pedunculated patch of the LHT grafted on the PJ anastomosis, the pancreatic stump, or site after tumor enucleation was used to prevent POPF. David A Iannitti et al. confirmed that the LHT as a vascular pedicle for reinforcing the pancreatic anastomoses results in a very low POPF rate during OPD [10]. And their wrapping technology is similar to our one, our experience also confirms that the simplicity of this technique, even during laparoscopic surgery. When only considering patients submitted to pancreatic anastomoses, there are only two related studies and data from low-evidence studies, but these studies indeed demonstrated the advantage in terms of reduced rate of POPF in the wrapping group [17, 19], which is consistent with our finding. Hassenpflug et al. reported their outcome that using the falciform ligament wrap pancreatic stump after distal pancreatectomy reduced the incidence of POPF, particularly of B and C grade, and thus resulted in a shorter hospital stay [20]. In our study, although there was no statistical significance. The mean postoperative hospital stay was shorter in wrapping group, which may be associated with the higher rate of POPF. Besides, Hackert et al. emphasized that the wrapping technology can significantly reduce POPF after tumor enucleation [21].

The oncological outcomes were similar between the two cohorts. The harvested lymph nodes and R0 resection rate were comparable between the two cohorts. these results confirm that the wrapping technique does not affect the R0 resection rate and the lymph node dissection range. However, our results are limited to a small sample size and short-term follow-up, further studies and long-term follow-up need to evaluate the oncological results.

It's also worth noting that complications related to the wrapping procedure. No complications directly related to the omental or falciform ligament flap, such as flap necrosis and infection, intestinal obstruction, perianastomotic collections and consequent intrabdominal abscesses were reported by these studies [17, 18], which is consistent with our finding.

These previously reported studies have obvious limitations, 1) All of these studies had a significant selection bias, and generally patients at high risk of POPF tend to wrap the anastomosis; 2) The mode of anastomosis, the proficiency of the surgeon, perioperative management were inconsistent in these studies; 3) Most of these studies were retrospective studies with small samples, consequently, these studies have the low methodological quality and the limitations of the available data; 4) There are also differences in the wrapping techniques in these studies. Our study effectively avoids many of the above limitations. All cases in this study were performed by the same surgeon, effectively avoiding inconsistency in anastomosis technique and wrapping technique. Second, the cases included in the non-wrapping cohort and wrapping cohort were completed in two different time periods to minimize selection bias. However, there are several limitations in this study, this study is still a relatively retrospective study with small samples. Although we introduced the PSM method to reduce confounding bias, the confounding variables could not be completely avoided. The results of this study still need a larger sample, well-designed randomized prospective studies for further validation.

## Conclusions

Wrapping the LTH around the PJ technique for LPD was safe, efficient, and reproducible with favorable perioperative outcomes in selected patients. As reported in previous studies, the technique could decrease the grade of severity of POPF. Therefore, rational application of this technique could help to increase the confidence of surgeon, especially beginners, and may potentially benefit selected patients at high risk of POPF. However, further validations using high-quality RCTs are still required to confirm the findings of this study.

### Supplementary Information

**Additional file 1. **

## Data Availability

The original contributions presented in the study are included in the article or supplementary material, and further inquiries can be directed to the corresponding author.
